# Altered amygdala structural connectivity and relations to social cognition in frontotemporal dementia

**DOI:** 10.1002/alz.71482

**Published:** 2026-05-18

**Authors:** Mengjie Huang, Marshall A. Dalton, Sophie Matis, Her Ai Claris Teng, Sau Chi Cheung, Yun T. Hwang, Rebekah M. Ahmed, Ramon Landin‐Romero, Olivier Piguet

**Affiliations:** ^1^ Brain and Mind Centre The University of Sydney Camperdown New South Wales Australia; ^2^ School of Psychology The University of Sydney Camperdown New South Wales Australia; ^3^ School of Health Sciences The University of Sydney Camperdown New South Wales Australia; ^4^ Neuropsychology Unit Royal Prince Alfred Hospital Camperdown New South Wales Australia; ^5^ Department of Neurology Gosford Hospital Gosford New South Wales Australia; ^6^ Central Coast Clinical School University of Newcastle Gosford New South Wales Australia; ^7^ Neurology Unit Royal Prince Alfred Hospital Camperdown New South Wales Australia

**Keywords:** amygdala, diffusion magnetic resonance imaging, fixel‐based analysis, frontotemporal dementia, social cognition, tractography

## Abstract

**INTRODUCTION:**

The amygdala is critical for social cognition and undergoes profound damage in frontotemporal dementia (FTD). While its atrophy is well documented, changes in its structural connectivity and their behavioral relevance remain unclear.

**METHODS:**

Using fixel‐based analysis and tractography, we examined amygdala connectivity in patients with behavioral variant FTD (bvFTD; *n* = 21), semantic dementia (SD; *n* = 19), progressive non‐fluent aphasia (PNFA; *n* = 18), and 28 controls. Associations with empathy and emotion recognition were explored using partial correlations.

**RESULTS:**

BvFTD and SD showed marked degeneration of amygdala‐associated tracts, while PNFA exhibited subtle left temporal changes. Tractography revealed reduced amygdala connectivity with regions supporting memory, visual, language, semantic, and motor functions, to varying degrees across subtypes. Social cognitive deficits were correlated with amygdala–cerebellum connectivity in bvFTD and with amygdala–hippocampus connectivity in SD.

**DISCUSSION:**

These findings were the first to demonstrate subtype‐specific patterns of amygdala white matter alteration and their relevance to social cognitive symptoms in FTD.

## Highlights

- We identify syndrome‐specific amygdala connectivity changes in FTD.
- Empathy loss in bvFTD and SD arise from distinct network disruptions.
- Amygdala white matter changes likely precede gray matter atrophy in progressive non‐fluent aphasia.

## 1 BACKGROUND

Frontotemporal dementia (FTD) is a leading cause of younger‐onset dementia, marked by progressive changes in behavior and/or language due to degeneration in frontal and temporal lobes.^1^ Clinically, FTD encompasses three variants: behavioral variant FTD (bvFTD) is characterized by prominent personality and behavioral changes,^2^ semantic dementia (SD) is associated with loss of conceptual knowledge,^3^ and progressive non‐fluent aphasia (PNFA) typically presents with impaired speech production.^4^ While these syndromes are traditionally associated with cortical atrophy, emerging research increasingly recognizes early subcortical involvement. Of particular interest is the amygdala, a small subcortical brain region located in the medial temporal lobes that plays a central role in emotion, memory, reward, and learning. ^5^

In contrast to the well‐documented amygdala atrophy in FTD,^6^, ^7^, ^8^, ^9^, ^10^ little is known about how its structural connectivity is affected. This represents an important gap in clinical knowledge, as the amygdala maintains extensive reciprocal connections with regions commonly implicated in FTD, such as the hippocampus and thalamus, which together support social cognitive functions. Social cognitive deficits, particularly impaired emotion recognition and empathy loss, are core clinical features of the disorder.^11^ Previous studies predominantly focused on gray matter integrity, showing that amygdala atrophy is associated with these symptoms,^12^, ^13^, ^14^, ^15^ far less is known about the contribution of white matter alterations. Given that disruption of amygdala pathways is likely to affect networks supporting social cognitive functions, characterizing its structural connectivity changes offers a promising avenue to understanding the neural basis of these symptoms in FTD syndromes.

Prior investigations of white matter changes in FTD mainly used whole‐brain approaches. These studies reported degeneration of tracts associated with the amygdala, particularly the inferior longitudinal fasciculus (ILF) and uncinate fasciculus in bvFTD and SD.^16^, ^17^, ^18^, ^19^, ^20^ In PNFA, degeneration is typically observed in language‐related pathways, notably the superior longitudinal fasciculus and the arcuate fasciculus,^16^, ^19^, ^21^, ^22^ whereas involvement of amygdala‐associated tracts was reported previously but not consistently.^16^, ^23^, ^24^, ^25^ Crucially, most of the prior work employed diffusion tensor imaging (DTI), which models a single dominant fiber orientation per voxel and does not account for crossing fibers that are present in up to 90% of imaging voxels in the adult brain.^26^, ^27^ This limitation is particularly relevant when examining amygdala‐associated pathways, as many them pass through regions of complex fiber architecture in the temporal lobes. Constrained spherical deconvolution (CSD) is a higher‐order diffusion model that addresses the crossing‐fiber problem by estimating the orientation and density of multiple fiber populations within each voxel.^28^ A novel statistical framework, known as fixel‐based analysis (FBA), applies these estimates to quantify the properties of individual fiber populations (i.e., fixels), thereby enabling fiber‐specific assessment of white matter integrity with greater biological specificity.^29^ As such, this approach offers greater sensitivity than DTI models in detecting subtle changes in regions with complex fiber configurations.

Taken together, the aim of this study was to investigate alterations in amygdala structural connectivity in the main FTD subtypes and explore their associations with social cognitive deficits. To address this, we first applied FBA to identify fiber‐specific alterations in major amygdala‐associated white matter tracts. We additionally identified the most highly connected brain regions of the amygdala using probabilistic tractography and quantitatively assessed how these connections varied across FTD subtypes and related to measures of empathy and emotion recognition. Consistent with known amygdala atrophy profiles, we hypothesized that bvFTD and SD would show the most pronounced degeneration of amygdala‐associated tracts compared to controls, with SD exhibiting a more asymmetric pattern. In the tractography analysis, we expected that the amygdala would show strongest connectivity with adjacent temporal structures (e.g., hippocampus and entorhinal cortex) and disrupted amygdala connectivity would correlate with social cognitive impairment in each FTD subtype.

## 2 METHODS

### 2.1 Participants

Fifty‐eight individuals diagnosed with FTD (21 bvFTD, 19 SD, 18 PNFA) and 28 healthy controls were recruited from FRONTIER, the FTD research clinic in Sydney, Australia, between March 2008 and February 2020. Diagnoses were made according to current consensus criteria,^2^, ^4^ following a comprehensive clinical, cognitive, and neuroimaging assessment, complemented by carer‐based questionnaires. The majority of participants (97%) had no significant family history of FTD. One patient carried a *GRN* mutation and a second a *C9orf72* repeat expansion, both were clinically diagnosed with bvFTD. Control participants were healthy volunteers, recruited from the relatives and friends of patients, as well as the broader community. All controls scored 88 or above out of 100 on Addenbrooke's Cognitive Examination Revised (ACE‐R) ^30^ or ACE‐III,^31^ indicating normal cognitive performance.

Patients and controls who had comorbid neurological or psychiatric conditions, a significant history of substance or alcohol abuse, limited English proficiency, or magnetic resonance imaging (MRI) contraindications were excluded from the study. SD patients with predominantly right‐lateralized temporal lobe atrophy were also excluded to ensure a homogeneous sample, as these cases often present with mixed behavioral and semantic deficits.

All participants gave written informed consent prior to assessment, in accordance with the Declaration of Helsinki. The study was approved by the South Eastern Sydney Local Health District and the Ethics Committees of the University of New South Wales and the University of Sydney.

### 2.2 Measures of cognitive function and disease severity

Global cognitive functioning was assessed in all participants using ACE‐R ^30^ or ACE‐III,^31^ which provided a combined measure of attention, memory, fluency, language, and visuospatial abilities. Where applicable, ACE‐R scores were converted to ACE‐III equivalents using our previously published conversion formula. ^32^

For the patient groups, the Frontotemporal Dementia Rating Scale (FRS) was used to evaluate functional impairment,^33^ where lower scores indicate increased functional decline. Clinical severity was also indexed using the Clinical Dementia Rating (CDR) scale,^34^ with higher scores indicating greater disease severity. Disease duration was calculated as the number of years from the initial onset of symptoms to the date of assessment.

**RESEARCH IN CONTEXT**

1. **Systematic review**: Review of the relevant literature indicates consistent amygdala atrophy across the canonical FTD syndromes. Yet little is known about alterations in amygdala structural connectivity and their relations to clinical symptoms.
2. **Interpretation**: We identify syndrome‐specific alterations in amygdala structural connectivity, affecting both major white matter tracts and direct region‐to‐region pathways. The evidence also supports the view that social cognitive impairments in bvFTD and semantic dementia arise from distinct neural mechanisms.
3. **Future directions**: Further research is needed to examine how amygdala network disruption evolves over time and its clinical relevance. Investigation of amygdala‐basal ganglia pathways may also shed light on motivational disturbances such as apathy, which are commonly observed across FTD syndromes.

### 2.3 Measures of social cognition

#### 2.3.1 Empathy

Empathy is conceptualized to encompass two components: cognitive empathy and affective empathy. Cognitive empathy refers to the capacity to understand others’ emotional state without necessarily sharing it (i.e., being able to put oneself in someone else's shoes); affective empathy refers to the capacity to share another person's emotional state (i.e., feeling what someone else is feeling).^35^ In the present study, these dimensions were assessed using the Perspective Taking and Empathic Concern subscales of the Interpersonal Reactivity Index (IRI).^36^ Each subscale included seven items rated on a 5‐point Likert scale (0 = “*does not describe well*” to 4 = “*describes very well*”), with a maximum score of 28. The raw scores were converted to percentages for statistical analysis. In healthy controls, the IRI was completed by self‐report, while for patients, ratings were provided by their carer.

#### 2.3.2 Emotion recognition

Emotion recognition was assessed using the Face Affect Discrimination Task (FADT) and the Face Affect Selection Task (FAST).^37^, ^38^ Each task included 42 trials involving faces that expressed one of six primary emotions (i.e., happiness, sadness, anger, fear, disgust, and surprise) or a neutral expression. In the FADT, participants viewed pairs of faces and were asked to judge whether the emotional expressions were the same or different. In the FAST, they were shown an array of seven faces from the same individual and asked to select the face matching a verbal label (e.g., “*point to the angry face*”).

As basic face perception may influence performance on emotion recognition tasks, the Face Perception Task (FPT) was administered to assess lower‐level perceptual processing.^38^ In this task, participants viewed 40 pairs of faces displaying neutral expressions and indicated whether the faces in each pair were identical or not.

All the tasks were scored by awarding one point per correct response, and accuracy was calculated as a percentage. Feedback was not provided during the tasks.

### 2.4 Image acquisition

Diffusion‐ and T1‐weighted images were acquired within 3 months of clinical assessment using two 3T magnetic resonance imaging (MRI) scanners equipped with standard eight‐channel head coils. Most participants (*n* = 85; 92%) were scanned on a Philips 3T scanner, with a small subset (*n* = 7; 8%) scanned on a GE Discovery MR750 scanner using harmonized acquisition protocols. For the Philips 3T scanner, whole‐brain diffusion‐weighted echo‐planar images were acquired with 32 non‐collinear gradient directions: repetition time = 8400 ms, echo time = 68 ms, 55 horizontal slices, voxel resolution = 2.5 × 2.5 × 2.5 mm, field of view = 240 × 240 mm, acquisition matrix = 96 × 96, flip angle = 90. One image without diffusion weighting (*b* = 0 s/mm^2^) was included. For the GE Discovery MR750 scanner, diffusion‐weighted images were acquired with 64 non‐collinear gradient directions: repetition time = 6000 ms, echo time = 60 ms, 60 horizontal slices, voxel resolution = 0.9375 × 0.9375 × 2.5 mm, field of view = 256 × 256 mm, and acquisition matrix = 256 × 256, flip angle = 90. Six *b* = 0 s/mm^2^ images were included. To minimize inter‐scanner variability, key diffusion‐weighted imaging (DWI) parameters, such as the use of a single‐shell protocol with a *b*‐value of 1000 s/mm^2^, were maintained across scanners.

For T1‐weighted data, the images were acquired with the following parameters on both scanners: repetition time = 5.8 ms, echo time = 2.6 ms, in‐plane matrix = 256 × 256, 200 slices, slice thickness = 1 mm, isotropic voxel resolution, flip angle α = 8.

### 2.5 Neuroimaging analysis

To confirm the topography of each syndrome, a cortical thickness analysis was performed comparing each FTD group with controls. The methods and results of this analysis are presented in the Supplementary Material, with the patterns of cortical thinning in each group shown in Figure S1.

**FIGURE 1:**
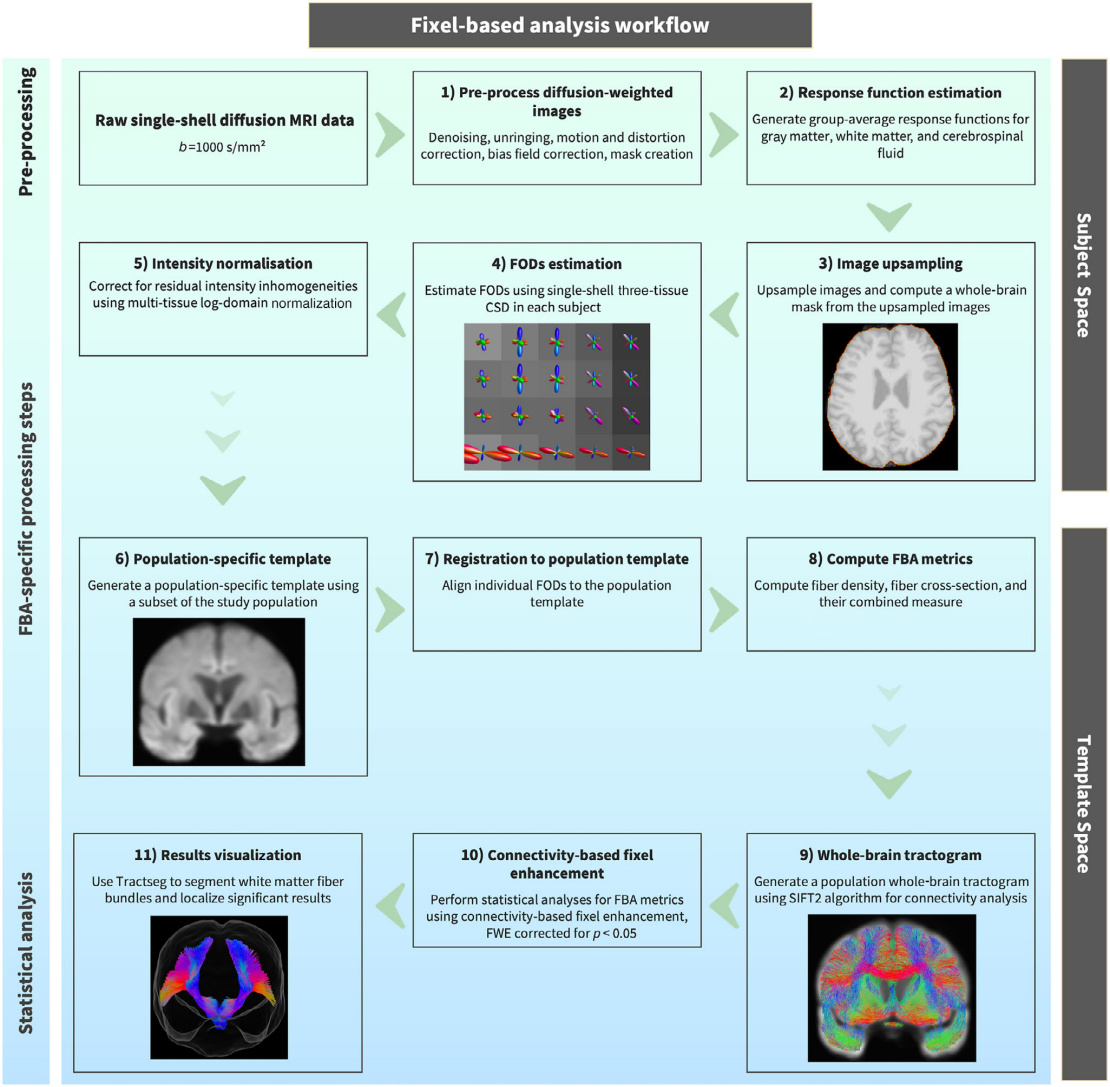
Fixel‐based analysis workflow. CSD, constrained spherical deconvolution; FBA, fixel‐based analysis; FOD, fiber orientation distribution; FWE, family‐wise error; SIFT, spherical‐deconvolution informed filtering of tractograms.

**FIGURE 2:**
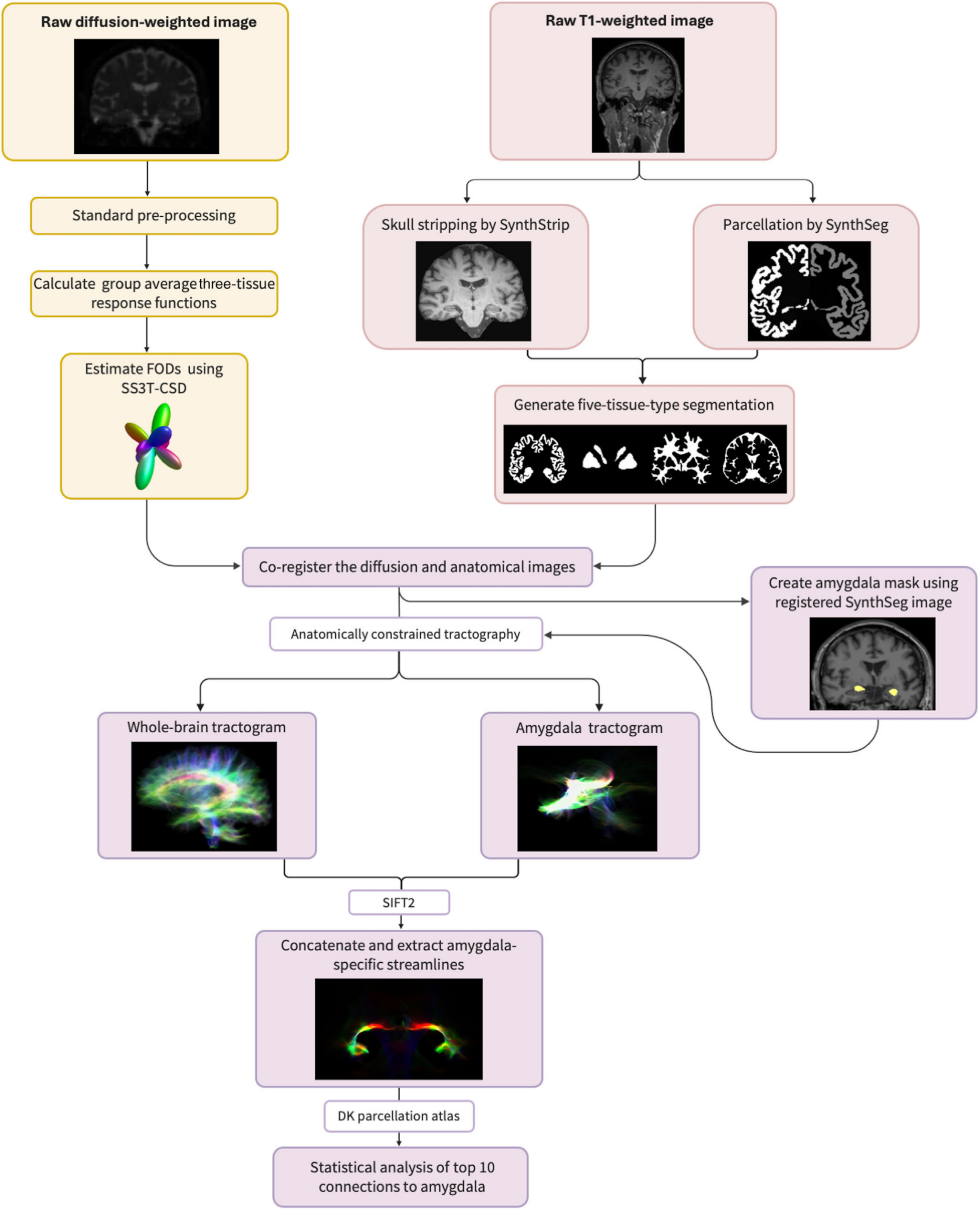
Tractography workflow. DK, Desikan‐Killiany; FODs, fiber orientation distributions; SIFT, spherical‐deconvolution informed filtering of tractograms; SS3T‐CSD, single‐shell three‐tissue constrained spherical deconvolution.

Fixel‐based and tractography analyses were conducted using MRtrix3 version 3.0.1 (https://www.mrtrix.org/).^39^ Other software packages, including FSL (https://fsl.fmrib.ox.ac.uk/fsl/) and FreeSurfer (https://surfer.nmr.mgh.harvard.edu/), were also routinely incorporated into the analysis pipeline. The full workflow of both analyses is illustrated in Figure 1 and Figure 2, respectively.

#### 2.5.1 FBA

The diffusion‐weighted images were first pre‐processed following the recommended pipeline for single‐shell diffusion MRI data, including denoising, Gibbs ringing correction, motion and distortion correction, bias field correction, and brain masking to minimize artifacts. Tissue response functions for white matter, gray matter, and cerebrospinal fluid were computed for each participant and averaged to produce group response functions. Images were upsampled to 1.25‐mm voxel resolution. Next, fiber orientation distributions (FODs) were computed in each participant using the single‐shell three‐tissue CSD (SS3T‐CSD) method,^40^, ^41^ followed by intensity normalization across tissue types. A study‐specific FOD template was then generated from a subset of 28 participants (seven per group), to which all individual FOD maps were non‐linearly registered. Fixels were segmented from the template using a peak amplitude threshold of 0.08. Three fiber‐specific metrics were calculated: fiber density (FD), fiber cross‐section (FC), and their combined measure, fiber density and cross‐section (FDC), providing information on white matter micro‐ and macrostructure properties.^42^ A whole‐brain probabilistic tractogram was then generated from the FOD template and filtered using the spherical‐deconvolution informed filtering of tractograms (SIFT2) algorithm.^43^ A total of 20 million streamlines were generated and filtered to two million to reduce biases in tractogram densities.

#### 2.5.2 Tractography

Tractography was performed using a region of interest (ROI)‐based approach, with the amygdala employed as the seed region for reconstructing streamlines. Diffusion‐weighted images were first pre‐processed following the same steps for FBA as previously described, and FODs were computed using the SS3T‐CSD method for all participants. In parallel, the corresponding T1‐weighted images were processed using the SynthSeg ^44^ and SynthStrip ^45^ tools in FreeSurfer version 7.4.1, to provide automated brain segmentation and skull stripping, respectively. From the SynthSeg outputs, five‐tissue‐type (5TT) files were generated, which comprised five volumes corresponding to cortical gray matter, subcortical gray matter, white matter, cerebrospinal fluid, and pathological tissue. All 5TT images were visually inspected to ensure accurate delineation of tissue boundaries. Next, the anatomical and diffusion‐weighted images were co‐registered using rigid‐body transformation to bring them into spatial alignment. Amygdala masks were derived from the registered SynthSeg segmentation and manually corrected in MRview.

Probabilistic tractography was then performed using the iFOD2 algorithm. Ten million whole‐brain streamlines and an additional 10 million amygdala‐seeded streamlines were generated per subject at 0.2‐mm isotropic resolution. The streamlines were filtered using the SIFT2 algorithm,^46^, ^47^ which assigned biologically informed weights to each streamline. In line with previous work,^48^ streamlines terminating in the amygdala were extracted along with their SIFT2 weights to isolate amygdala‐specific connections. A structural connectome was generated for each subject. Brain regions were defined using the Desikan‐Killiany atlas,^49^ with the automated amygdala segmentation replaced by the manually edited mask for greater anatomical accuracy. Connectivity strength between the amygdala and each brain region was quantified as the sum of SIFT2 weights, with higher weights indicating greater connectivity strength. For each participant, left and right hemisphere connections were combined to yield bilateral connectivity values. These values were then averaged across the study sample, and the 10 brain regions with the highest mean connectivity to the amygdala were identified for subsequent group comparisons.

### 2.6 Statistical analyses

Group comparisons of fixel‐based metrics were conducted using a general linear model in MRtrix3. FDC was used as the primary metric in the present study, as it integrates both microstructural and macrostructural white matter properties while reducing the need for multiple comparisons when testing FD and FC separately. Statistical inference was performed using connectivity‐based fixel enhancement, which incorporates structural connectivity information from the template tractogram.^50^ Non‐parametric permutation testing was applied with 5000 permutations; statistical significance was set at family‐wise error corrected *p* < 0.05.

**FIGURE 3:**
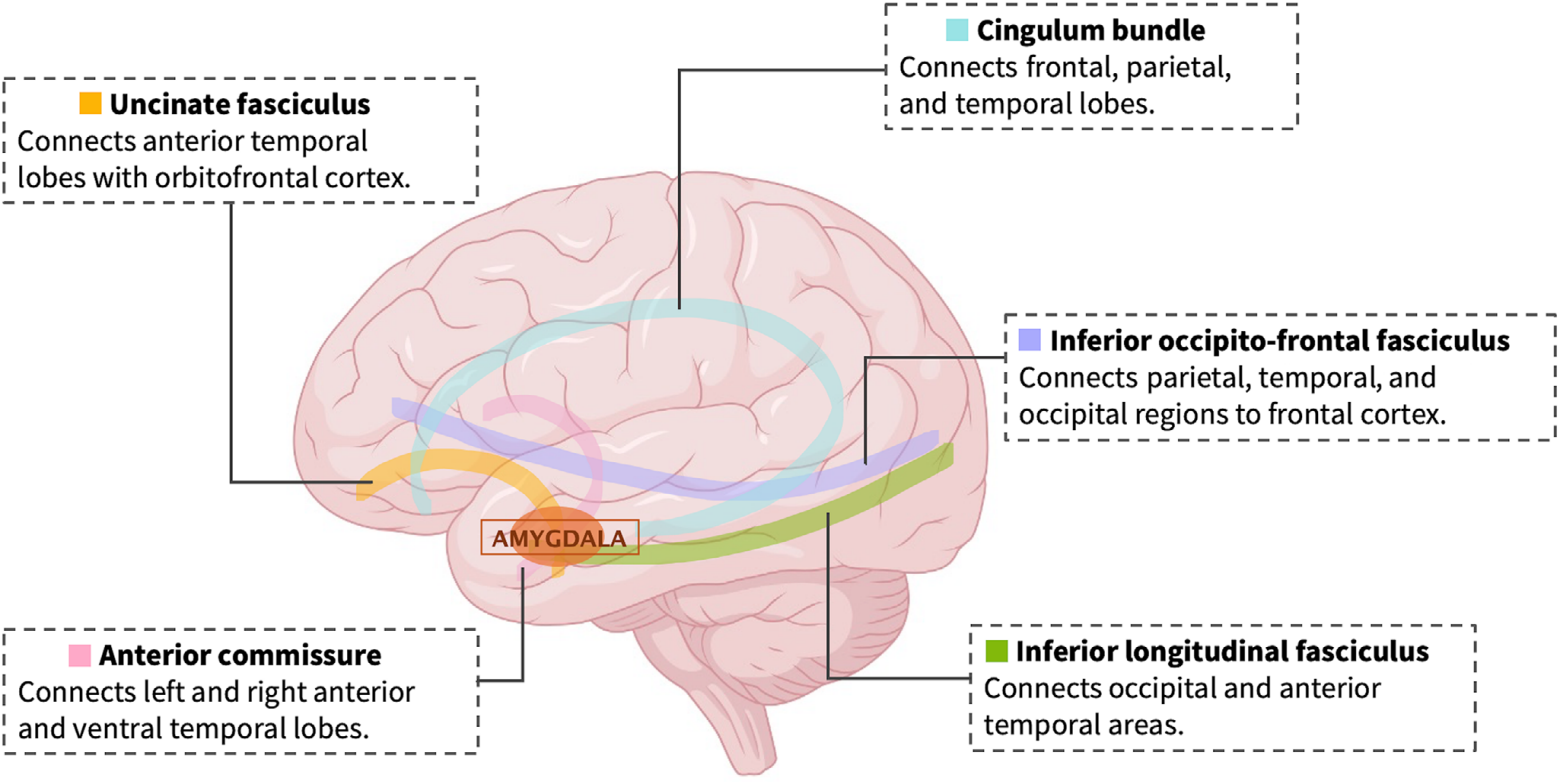
White matter tracts of interest. Schematic illustration of the five major amygdala‐associated white matter tracts examined in the fixel‐based analysis.

Significant fixels were localized to white matter tracts using TractSeg.^51^ FDC was then compared across five major amygdala‐associated tracts (Figure 3), selected a priori based on existing literature,^52^ including the uncinate fasciculus,^53^ anterior commissure,^54^ cingulum bundle,^55^ ILF,^56^ and inferior fronto‐occipital fasciculus (IFOF).^57^, ^58^ To aid interpretation, effect sizes were expressed as percentage differences relative to the control group mean using the following formula: (Group mean − Control mean)/Control mean × 100%.

All other statistical analyses were performed in SPSS version 29.0 (IBM SPSS Statistics); visual representations were generated with GraphPad Prism version 10.3.0 (GraphPad Software, Boston, MA, USA). Continuous variables were first tested for normality and homogeneity of variance using the Shapiro‐Wilk and Levene's tests, respectively. Parametric data were analyzed using ANOVA with Sidak correction for post hoc tests. For non‐parametric variables, the Kruskal‐Wallis test was employed, followed by Mann‐Whitney U tests for pairwise comparisons where applicable. Group differences in emotion recognition tasks (FADT and FAST) were assessed using multivariate analysis of covariance (MANCOVA), adjusting for FPT performance. Categorical variables (e.g., sex) were assessed using chi‐squared tests.

Finally, partial Spearman correlations, corrected for multiple comparisons (*p* < 0.01), were performed within each FTD group to assess the association between amygdala connectivity strength (indexed by SIFT2 weights) and social cognitive performance. Analyses were restricted to amygdala connections showing significant reductions compared to controls to minimize Type I error. All models were adjusted for global cognition and disease severity. For emotion recognition tasks, FPT performance was included as an additional covariate to account for lower‐level perceptual abilities.

## 3 RESULTS

### 3.1 Demographic and clinical characteristics

Groups were matched for age, sex, and years of education (Table 1) and disease duration was similar across the FTD subtypes. In terms of cognitive function, significant differences were observed in ACE‐III total scores, with all patient groups performing significantly worse than healthy controls (*p* < 0.003 for all comparisons). Among the patient groups, SD exhibited greater cognitive impairment compared to bvFTD (*p* = 0.048) and PNFA (*p* < 0.001), reflecting the high language load of the task.

**TABLE 1:** Demographic characteristics and social cognitive test performance of the study samples.

	bvFTD	SD	PNFA	HC	Group effect	*p* value	Post hoc tests
	*n* = 21	*n* = 19	*n* = 18	*n* = 28			
Age (years)	61.8 ± 7.6	63.5 ± 6.9	64.4 ± 11.0	64.5 ± 6.5	0.454	ns	–
Sex (Male: Female)	14:7	12:7	9:9	14:14	2.014^a^	ns	–
Education (Years)	11.8 ± 2.8	12.0 ± 3.1	12.7 ± 2.7	13.4 ± 2.1	1.696	ns	–
Disease duration (Years)	4.3 ± 2.4	4.1 ± 1.5	3.4 ± 2.1	–	0.988	ns	–
ACE‐III total score (max: 100)	72.4 ± 18.3	61.9 ± 13.1	80.5 ± 9.1	93.9 ± 4.4	28.435	<0.001	bvFTD^**^, SD^**^, PNFA^*^ < HC; SD < bvFTD^*^, PNFA^**^
FRS Rasch total score	−0.3 ± 1.4	1.7 ± 1.3	2.5 ± 0.9	–	24.959	<0.001	bvFTD < SD^**^, PNFA^**^
CDR (sum of boxes; range: 0 to 18)	5.7 ± 2.8	2.6 ± 1.3	1.4 ± 1.3	–	24.459	<0.001	SD^**^, PNFA^**^ < bvFTD
Interpersonal Reactivity Index (%)							
Perspective taking	31.3 ± 17.9	38.4 ± 27.0	56.5 ± 22.5	65.1 ± 13.7	6.918	<0.001	bvFTD^*^, SD^*^ < HC; bvFTD^*^ < PNFA
Empathic concern	54.2 ± 15.8	61.4 ± 22.1	66.0 ± 22.4	78.6 ± 15.8	2.954	0.041	bvFTD^*^ < HC
Emotion Recognition Tests (%)							
Face perception	92.8 ± 10.0	95.7 ± 8.7	95.8 ± 5.5	98.9 ± 2.0	7.881^b^	0.049	–
Face affect discrimination	77.9 ± 13.2	83.2 ± 9.1	86.6 ± 7.7	90.8 ± 4.3	18.908^c^	<0.001	bvFTD^**^, SD^*^ < HC
Fast affect selection	65.0 ± 21.4	73.3 ± 12.3	85.4 ± 10.5	92.2 ± 4.2	37.865^c^	<0.001	bvFTD^**^, SD^**^ < HC; bvFTD^*^ < PNFA

*Note*: Values are mean ± standard deviation. Missing data: Clinical Dementia Rating Scale: 1bvFTD, 1SD; Interpersonal Reactivity Index: 5bvFTD, 3SD, 3PNFA, 19Controls; Emotion Recognition Tests: 2bvFTD, 1SD, 2PNFA, 2Controls.

Abbreviations: ACE, Addenbrooke's cognitive examination; bvFTD, behavioral‐variant frontotemporal dementia; CDR, Clinical Dementia Rating scale; FRS, frontotemporal dementia rating scale; HC, healthy controls; ns, non‐significant; PNFA, progressive non‐fluent aphasia; SD, semantic dementia.

^a^ Chi‐squared test.

^b^ Kruskal‐Wallis H test.

^c^ MANCOVA test.

^*^ *p* < 0.05; **p < 0.001.

Disease severity also differed significantly among the FTD groups. Patients with bvFTD showed lower FRS and higher CDR scores compared to those with SD and PNFA (all *p* values < 0.001), indicating greater disease severity and functional impairment in this group.

### 3.2 Neuroimaging results

#### 3.2.1 FBA

**FIGURE 4:**
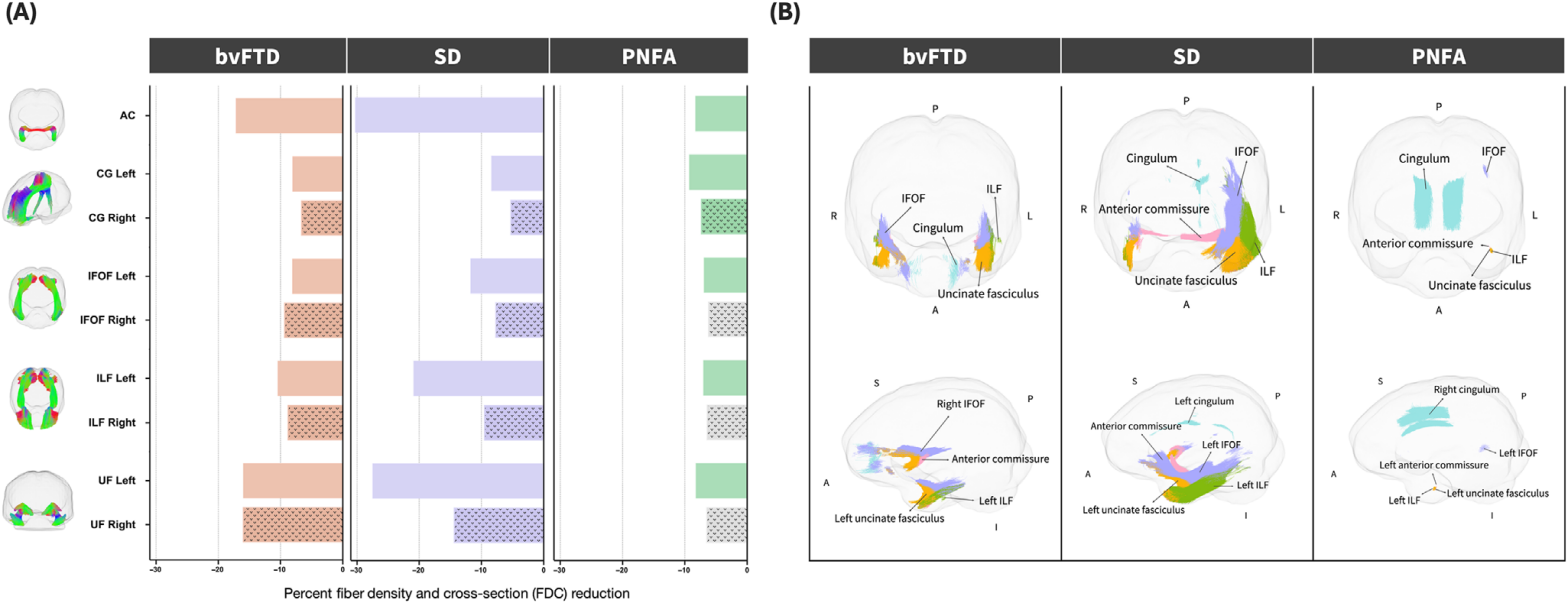
Amygdala‐associated white matter tract degeneration across FTD subtypes. (A) Percent FDC reduction in the five amygdala‐associated white matter tracts in FTD groups compared with controls. Gray bars represent differences that did not reach statistical significance (set at family‐wise error corrected *p* < 0.05). (B) Glass brains visualization showing the significantly affected portion of the amygdala‐associated tracts in each FTD subtype. A, anterior; AC, anterior commissure; bvFTD, behavioral variant frontotemporal dementia; CG, cingulum bundle; FDC, fiber density and cross‐section; I, inferior; IFOF, inferior occipito‐frontal fasciculus; ILF, inferior longitudinal fasciculus; L, left; P, posterior; PNFA, progressive non‐fluent aphasia; R, right; S, superior; SD, semantic dementia; UF, uncinate fascicle.

FBA results of the amygdala‐associated tracts are presented in Figure 4 and Figure S2. BvFTD exhibited significant bilateral white matter degeneration compared to the controls. This was most pronounced in the anterior commissure (−17%) and uncinate fasciculus (left and right: −16%). Significant FDC reductions were also identified in the ILF (left: −10%, right: −9%), IFOF (left: −8%, right: −9%), and cingulum bundle (left: −8%, right: −7%).

SD showed the most pronounced and left‐lateralized FDC reductions across all amygdala‐associated tracts. The anterior commissure showed the greatest reduction (−30%), followed by the uncinate fasciculus (left: −28%, right: −14%). Substantial reductions were also observed in the ILF (left: −21%, right: −10%) and IFOF (left: −12%, right: −8%). Relatively less severe yet asymmetrical damage was observed in the cingulum bundle (left: −8%, right: −5%).

PNFA was affected to a lesser extent relative to the other two FTD subtypes; white matter reductions were mainly observed in the frontal regions. Significant FDC reductions were identified in the anterior commissure (−8%), cingulum bundle (left: −9%, right: −7%), left uncinate fasciculus (−8%), left ILF and IFOF (−7% for both tracts). Reductions in FDC were also found in the right hemisphere, compared with controls: uncinate fasciculus (−7%), ILF (−6%), and IFOF (−6%). These differences, however, were not statistically significant.

#### 3.2.2 Tractography

**FIGURE 5:**
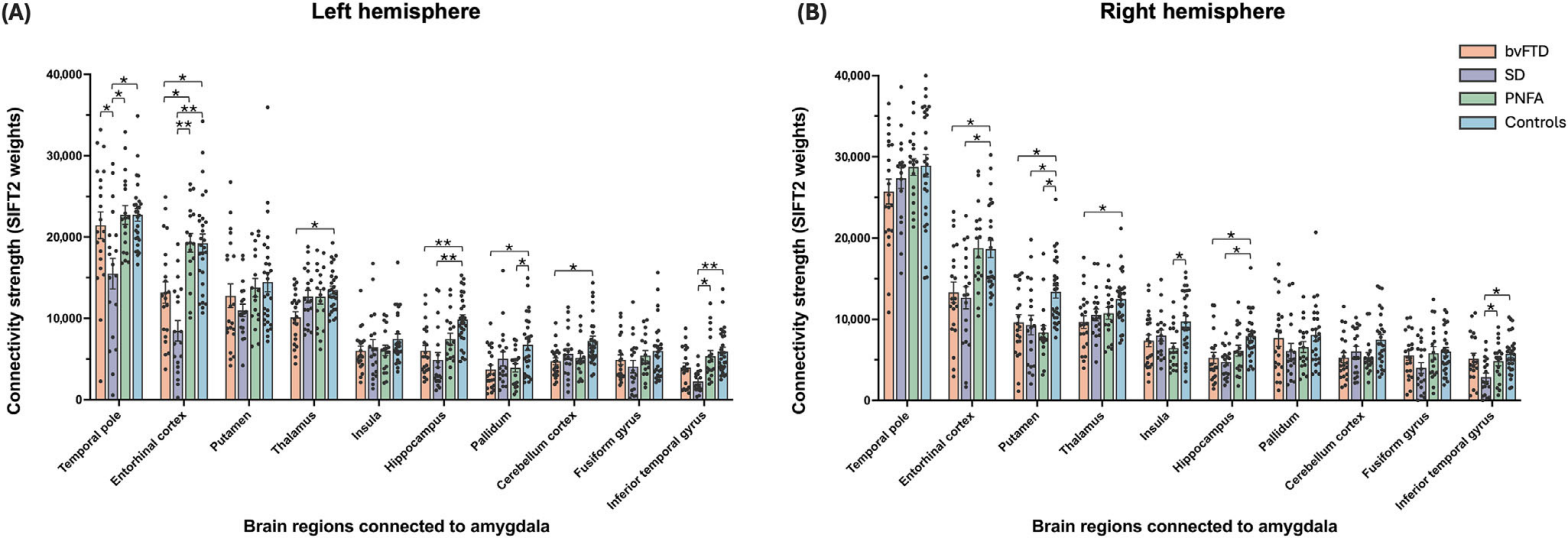
Group comparisons of connectivity strengths between the amygdala and its top 10 connected brain regions. Each bar represents the mean SIFT2 weights for each group, error bars indicate standard error of the mean, and the dots represent individual data points; *p < 0.05; **p < 0.001. bvFTD, behavioral variant frontotemporal dementia; PNFA, progressive non‐fluent aphasia; SD, semantic dementia.

The 10 regions with the strongest connectivity to the amygdala included the temporal pole, entorhinal cortex, putamen, thalamus, insula, hippocampus, pallidum, cerebellum, fusiform gyrus, and inferior temporal gyrus. Significant group differences were observed in all regions except in the fusiform gyrus. Group comparisons of these top 10 amygdala connections are presented in Figure 5, and the full statistics results (including means, standard deviations, and 95% confidence intervals) are summarized in Table S1.

Compared with controls, patients with bvFTD exhibited reduced connectivity strength between the amygdala and the bilateral entorhinal cortex (left: *p* = 0.020, right: *p* = 0.043), thalamus (left: *p =* 0.006, right: *p =* 0.035), hippocampus (left: *p <* 0.001, right: *p =* 0.006), left cerebellum (*p* = 0.009), left pallidum (*p* = 0.012), and right putamen (*p* = 0.031). In addition, connectivity with the left inferior temporal gyrus (*p* = 0.051) and right cerebellum (*p* = 0.056) also showed marginal significance. In the SD group, reduced connectivity strength was observed between the amygdala and the bilateral entorhinal cortex (left: *p <* 0.001, right: *p* = 0.024), hippocampus (left: *p* < 0.001, right: *p* = 0.001), inferior temporal gyrus (left: *p* < 0.001, right: *p* = 0.003), left temporal pole (*p* = 0.010), and right putamen (*p* = 0.023) compared to controls. In the PNFA group, patients showed reduced connectivity strength between the amygdala and the right insula (*p* = 0.017), right putamen (*p* = 0.003), and left pallidum (*p* = 0.033) compared to controls.

Across the FTD groups, SD exhibited the lowest connectivity strength between the amygdala and the left temporal pole compared to both bvFTD (*p* = 0.037) and PNFA (*p* = 0.014). SD also showed reduced connectivity between the amygdala and the left inferior temporal gyrus compared to PNFA (*p* = 0.002) and in the right inferior temporal gyrus compared to bvFTD (*p* = 0.015). Both bvFTD (*p* = 0.043) and SD (*p* < 0.001) demonstrated lower connectivity strength between the amygdala and the left entorhinal cortex compared to PNFA.

### 3.3 Social cognitive performance results

#### 3.3.1 Empathy

Significant group differences were observed for both Perspective Taking (*p* < 0.001) and Empathic Concern (*p* = 0.041) (Table 1). Post hoc analyses further indicated that bvFTD scored significantly lower than healthy controls on both subscales (Perspective Taking: *p* = 0.002; Empathic Concern: *p* = 0.032), whereas SD scored significantly lower only on Perspective Taking (*p* = 0.025). In contrast, no significant differences were found between PNFA and controls (both *p* values > 0.581). Finally, bvFTD scored significantly lower than PNFA on Perspective Taking (*p* = 0.010). No other comparisons between patient groups reached statistical significance.

#### 3.3.2 Emotion recognition

Significant group differences were observed for both emotion recognition tasks (both *p* values < 0.001), as well as for the FPT (*p* = 0.049). After adjusting for FPT performance, bvFTD were significantly impaired compared to controls on both FADT and FAST (*p* < 0.001 for both) and also performed significantly worse than PNFA on the FAST (*p* = 0.012). Finally, a trend toward impaired face perception in bvFTD relative to controls was noted (*p* = 0.058). SD also exhibited significant deficits compared to controls on both FADT (*p* = 0.020) and FAST (*p* < 0.001), as well as marginally lower scores compared to PNFA on the FADT (*p* = 0.051). In contrast, patients with PNFA did not significantly differ from controls on any of the emotion recognition measures (all *p* values > 0.5).

### 3.4 Correlations between amygdala connectivity strength and social cognition

In the bvFTD group, a significant positive correlation was observed between Perspective Taking and amygdala–left cerebellum connectivity (*r* = 0.713, *p* = 0.002), after controlling for general cognitive functioning and disease severity. Several noteworthy associations also emerged at a trend level. Specifically, Empathic Concern was positively correlated with amygdala‐left cerebellum connectivity (*r* = 0.512, *p* = 0.037), and Perspective Taking was positively associated with amygdala–right cerebellum connectivity (*r* = 0.508, *p* = 0.032). Regarding emotion recognition measures, a trend was observed between FADT and amygdala‐right cerebellum connectivity (*r* = 0.422, *p* = 0.052). A positive correlation was also found between FAST and amygdala‐left thalamus connectivity (*r* = 0.442, *p* = 0.038), after adjusting for disease severity, global cognition, and face perception ability.

In the SD group, amygdala–right hippocampus connectivity was significantly correlated with Perspective Taking (*r* = 0.634, *p* = 0.004) and FADT (*r* = 0.650, *p* = 0.006), after controlling for disease severity, cognitive functioning, and face perception ability. A trend was also found between amygdala–left entorhinal connectivity and Empathic Concern (*r* = 0.486, *p* = 0.046), though this did not reach the adjusted significance threshold.

No significant associations between amygdala structural connectivity and social cognitive measures were found in the PNFA group. Full statistical results are presented in Table 2.

**TABLE 2:** Partial correlations between significantly reduced amygdala connectivity and social cognitive test performance in the FTD groups.

	bvFTD				SD				PNFA			
	IRI_PT	IRI_EC	FADT	FAST	IRI_PT	IRI_EC	FADT	FAST	IRI_PT	IRI_EC	FADT	FAST
**Temporal pole**												
Left	–	–	–	–	0.088	0.417	0.029	0.330	–	–	–	–
					(−0.387, 0.526)	(−0.052, 0.735)	(−0.418, 0.465)	(−0.131, 0.674)				
Right	–	–	–	–	–	–	–	–	–	–	–	–
**Entorhinal cortex**												
Left	−0.414	−0.385	0.125	0.236	0.319	**0.486** ^*^	−0.108	0.189	–	–	–	–
	(−0.734, 0.055)	(−0.717, 0.090)	(−0.319, 0.524)	(−0.212, 0.602)	(−0.164, 0.679)	(0.035, 0.773)	(−0.525, 0.351)	(−0.276, 0.582)				
Right	−0.466	−0.137	−0.106	−0.345	0.003	−0.236	−0.033	−0.173	–	–	–	–
	(−0.780, 0.037)	(−0.561, 0.344)	(−0.510, 0.336)	(−0.673, 0.096)	(−0.457, 0.461)	(−0.627, 0.250)	(−0.468, 0.415)	(−0.571, 0.291)				
**Putamen**												
Left	–	–	–	–	–	–	–	–	–	–	–	–
Right	−0.055	−0.168	−0.170	0.001	−0.058	−0.269	−0.246	−0.012	−0.078	0.173	−0.118	−0.117
	(−0.501, 0.414)	(−0.582, 0.315)	(−0.557, 0.277)	(−0.426, 0.428)	(−0.504, 0.412)	(−0.648, 0.217)	(−0.621, 0.220)	(−0.452, 0.432)	(−0.536, 0.415)	(−0.332, 0.601)	(−0.568, 0.387)	(−0.568, 0.388)
**Thalamus**												
Left	−0.269	0.029	0.080	**0.442** ^*^	–	–	–	–	–	–	–	–
	(−0.648, 0.217)	(−0.436, 0.482)	(−0.359, 0.490)	(0.019, 0.731)								
Right	0.100	0.319	−0.269	0.116	–	–	–	–	–	–	–	–
	(−0.376, 0.534)	(−0.164, 0.679)	(−0.624, 0.178)	(−0.327, 0.517)								
**Insula**												
Left	–	–	–	–	–	–	–	–	–	–	–	–
Right	–	–	–	–	–	–	–	–	−0.038	0.005	0.460	−0.315
									(−0.507, 0.448)	(−0.474, 0.482)	(−0.076, 0.790)	(−0.689, 0.190)
**Hippocampus**												
Left	−0.426	−0.209	−0.138	0.025	0.097	0.297	0.213	−0.177	–	–	–	–
	(−0.740, 0.041)	(−0.609, 0.276)	(−0.534, 0.307)	(−0.406, 0.447)	(−0.379, 0.532)	(−0.187, 0.665)	(−0.253, 0.599)	(−0.574, 0.288)				
Right	−0.193	−0.359	0.301	0.262	**0.634** ^**^	0.306	**0.650** ^**^	−0.002	–	–	–	–
	(−0.599, 0.292)	(−0.702, 0.120)	(−0.145, 0.645)	(−0.186, 0.620)	(0.247, 0.847)	(−0.178, 0.671)	(0.292, 0.848)	(−0.444, 0.440)				
**Pallidum**												
Left	0.017	0.044	−0.043	0.003	–	–	–	–	0.258	0.328	0.350	0.223
	(−0.445, 0.472)	(−0.423, 0.493)	(−0.462, 0.391)	(−0.493, 0.498)					(−0.251, 0.655)	(−0.178, 0.697)	(−0.148, 0.711)	(−0.280, 0.639)
Right	–	–	–	–	–	–	–	–	–	–	–	–
**Cerebellum cortex**												
Left	**0.713** ^**^	**0.512** ^*^	−0.107	−0.222	–	–	–	–	–	–	–	–
	(0.378, 0.883)	(0.069, 0.786)	(−0.511, 0.335)	(−0.593, 0.226)								
Right	**0.508** ^*^	0.153	0.422	0.102	–	–	–	–	–	–	–	–
	(0.064, 0.784)	(−0.329, 0.572)	(−0.006, 0.719)	(−0.340, 0.507)								
**Fusiform gyrus**												
Left	–	–	–	–	–	–	–	–	–	–	–	–
Right	–	–	–	–	–	–	–	–	–	–	–	–
**Inferior temporal gyrus**												
Left	−0.143	−0.074	0.184	−0.094	−0.064	−0.156	0.190	−0.116	–	–	–	–
	(−0.565, 0.338)	(−0.515, 0.398)	(−0.264, 0.566)	(−0.501, 0.347)	(−0.508, 0.407)	(−0.574, 0.326)	(−0.275, 0.583)	(−0.531, 0.344)				
Right	–	–	–	–	−0.167	−0.264	−0.240	−0.218	–	–	–	–
					(−0.581, 0.316)	(−0.645, 0.222)	(−0.617, 0.226)	(−0.602, 0.248)				

*Note*: Analyses were restricted to amygdala connections showing significant reductions compared to controls to minimize Type I error.

Spearman partial correlation coefficients between amygdala connectivity (SIFT2 weights) and social cognitive measures in the FTD groups, controlling for cognition and disease severity. FADT and FAST were additionally adjusted for FPT performance. Values are correlation coefficients (90% confidence intervals).

Abbreviations: bvFTD, behavioral‐variant frontotemporal dementia; FADT, Facial Affect Discrimination Task; FAST, Facial Affect Selection Task; IRI_EC, Interpersonal Reactivity Index_Empathic Concern; IRI_PT, Interpersonal Reactivity Index_Perspective Taking; PNFA, progressive non‐fluent aphasia; SD, semantic dementia.

^*^ *p* < 0.05; **p < 0.01 (one‐tailed).

## 4 DISCUSSION

Applying novel DWI techniques, we provided the first evidence that white matter pathways connecting the amygdala to cortical brain regions involved in visual, memory, language, semantic, and motor processing are differentially affected across FTD subtypes and that these alterations are linked to social cognitive deficits. Our findings extend existing knowledge about amygdala damage in FTD, provide novel insight into the neural basis of social cognitive impairment in FTD, potentially informing the development of more tailored interventions.

### 4.1 BvFTD

Consistent with the bilateral frontotemporal white matter degeneration in bvFTD,^16^, ^18^, ^19^ we observed symmetrical FDC reductions across all amygdala‐associated tracts, most prominently affecting the anterior commissure and uncinate fasciculus. This pattern was also reflected in the tractography results, which revealed widespread reduction in amygdala connectivity strengths. In particular, connections to the cerebellum and thalamus appear to be selectively affected in bvFTD, aligning with previous reports of greater cerebellar and thalamic involvement in this variant.^59^, ^60^, ^61^ Notably, both FBA and tractography results identified altered white matter pathways connecting the amygdala with nodes belonging to the salience network. The reduced functional integrity of this network is a key neural feature of bvFTD and has been consistently associated with social cognitive disturbances.^62^, ^63^ Here, our findings indicate that disruption of amygdala‐related pathways may provide a structural basis underlying these functional network changes.

Crucially, we identified a robust link between amygdala–cerebellum connectivity strength and empathic measures, implicating a circuit that has received little attention in the FTD literature. The cerebellum is increasingly recognized for its involvement in complex cognitive and social processes, and one proposed mechanism is via predictive processing, which refers to generating internal models to anticipate others’ intentions and guide socially adaptive responses.^64^, ^65^, ^66^ Given the role of the amygdala in detecting emotionally salient stimuli, reduced connectivity with the cerebellum may disrupt the integration of emotional information into these predictive processes, thereby affecting the ability to use emotional cues to guide social behaviors. Alternatively, disrupted amygdala–cerebellum communication may compromise the perceptual input that supports empathic responses. Emotion recognition, especially for negative expressions, is thought to be a key component of empathy.^67^ In the present study, patients with bvFTD showed marked deficits in emotion recognition, and reduced amygdala–right cerebellum connectivity was associated with poorer performance on the FADT, which requires perceptual matching of facial expressions. As the cerebellum supports rapid, automatic processing of emotional expressions ^68^ and the amygdala directs attention to salient emotional cues,^69^ disrupted communication between the two may interfere with early, bottom‐up perceptual processes that support empathic responses.

Another important finding was the correlation between reduced amygdala‐left thalamus connectivity and impaired performance on the FAST, which requires matching verbal emotion labels to facial expressions. While patients with bvFTD typically have preserved knowledge of emotion words, they often struggle to apply it to emotional cues.^37^ Our finding suggests that rather than a disruption in semantic knowledge, the deficit arises from a breakdown in the top‐down cognitive processes that support emotion interpretation. Crucially, the amygdala–thalamus pathway is thought to relay emotionally salient signals to prefrontal regions involved in higher‐order appraisal and regulation.^70^, ^71^ In this light, reduced connectivity of this pathway may impair the transmission processes, making it difficult for individuals to link what they experience with what they know. Taken together, our results suggest that amygdala connectivity alterations in bvFTD likely affect both early perceptual and higher‐order regulatory processes, which contribute to emotion recognition and empathy deficits observed in this group.

### 4.2 Semantic dementia

As expected, SD exhibited the most severe FDC reductions across all amygdala‐associated tracts, especially in the left hemisphere. The anterior commissure and the left uncinate fasciculus were most affected, followed by left ILF and IFOF. Notably, contrary to previous reports of early white matter changes confined to the left hemisphere,^16^ our results revealed significant FDC reductions in the right uncinate fasciculus, ILF, and IFOF, suggesting a more widespread pattern of white matter degeneration in SD than previously described. Tractography results also revealed widespread reductions in amygdala connectivity; reduced connectivity to the left temporal pole and bilateral inferior temporal gyrus were exclusive to this group.

In terms of behavioral associations, reduced amygdala–right hippocampus connectivity strength was correlated with both Perspective Taking and FADT scores, and a trend also emerged for amygdala–left entorhinal cortex connectivity and Empathic Concern. These associations point to the involvement of memory systems in empathy loss, as both hippocampus and entorhinal cortex support episodic and semantic memory and interact with the amygdala during emotional memory processing.^72^ Drawing on this framework, empathy is thought to rely on the ability to access and use past personal experiences when interpreting the emotional states of others.^73^ When connections between the amygdala and memory structures are disrupted, as observed in SD, this process is likely to be compromised, leading to difficulties in recognizing emotions and using memory to support empathic abilities.

Importantly, although reduced amygdala–hippocampus connectivity was also present in bvFTD, it was not significantly associated with empathy loss, indicating that empathy loss in bvFTD and SD arise from different network disruptions. Indeed, empathy is a multifaceted construct involving emotional, cognitive, and memory‐related processes.^73^ Our results suggest that in SD, empathy loss appears to be related to degraded emotional memory processes, whereas in bvFTD, it arises from difficulties using emotional cues to guide social behavior. Notably, impaired emotion recognition is likely to be a shared contributing factor across both groups. Taken together, these findings provide important evidence for distinct mechanisms underlying empathy loss in bvFTD and SD. Future task‐based functional imaging studies will be important to clarify these patterns.

### 4.3 Progressive non‐fluent aphasia

In contrast to bvFTD and SD, PNFA exhibited less severe amygdala white matter degeneration, with all tracts showing FDC reductions of less than 10% compared to controls. The most severely affected tracts were frontally located, specifically involving the bilateral cingulum bundle and a small posterior segment of the left IFOF. Importantly, FBA revealed statistically significant FDC reductions within the left temporal ILF and uncinate fasciculus, for which previous reports yielded mixed findings.^16^, ^23^, ^24^ This result likely reflects the greater sensitivity of FBA over traditional DTI models, owing to its capacity to resolve crossing fibers and detect subtle white matter changes that might be overlooked with voxel‐averaged approaches.

Notably, our previous work in PNFA patients with comparable demographic and clinical characteristics reported preserved amygdala volume in the early disease stage.^6^ The present findings nonetheless demonstrate alterations in amygdala structural connectivity in PNFA, suggesting that fiber‐specific white matter changes may emerge when gray matter involvement is not yet prominent. This finding holds important clinical relevance, as such early disruptions in connectivity can provide sensitive markers of disease progression and assist with diagnosis and staging in PNFA.

Supporting the FBA findings, tractography results also demonstrated less extensive yet significant alterations in amygdala connectivity in PNFA, specifically involving the right insula, putamen, and left pallidum. These altered connections, however, were not related to social cognitive performance, nor did PNFA patients exhibit significant decline in these measures. This pattern is in keeping with evidence that social cognitive impairments are less pronounced in this group and are not related to amygdala connectivity changes in the early disease stage. The altered connections instead involved regions integral to motor control, interoception, and speech production, suggesting that amygdala connectivity disruption in PNFA may hold greater relevance for these functions. For instance, reduced connectivity with basal ganglia structures may affect the integration of emotional and motivational cues into motor behavior, potentially contributing to reduced motivation or apathy. Although apathy was not assessed in this study, it has been reported in PNFA,^74^ suggesting a potential link worthy of further investigation. Moreover, these connectivity alterations may reflect early structural changes preceding more pronounced behavioral and emotional disturbances that emerge as the disease progresses.^75^

### 4.4 Limitations

Several limitations should be noted. First, while our findings provide evidence for the relevance of amygdala structural connectivity to social cognitive symptoms in FTD, results should be interpreted with caution due to the small group size. Second, the absence of a standardized measure of behavioral disturbances limits our understanding of how the observed changes relate to neuropsychiatric symptoms such as apathy. Future studies that incorporate larger samples and include behavioral disturbance scales are needed to confirm these preliminary findings and to better characterize the role of amygdala alterations in FTD. Third, the cross‐sectional design precluded examination of how amygdala connectivity evolves with disease progression. Longitudinal studies will help elucidate the dynamic unfolding between gray and white matter changes affecting the amygdala and their relation to social functioning in the canonical FTD syndromes. It is also noteworthy that right‐lateralized SD patients were excluded to ensure a homogeneous sample. Future work directly contrasting left and right SD subtypes will be essential for understanding how laterality influences amygdala connectivity profiles. Moreover, we did not attempt to map structural connectivity patterns of individual amygdala nuclei due to limited spatial resolution, an issue that will require ultra‐high‐resolution scanning acquisition and advanced segmentation tools to assess nucleus‐specific connectivity patterns. Lastly, our data‐driven approach focused on the 10 most relevant pathways but may have excluded other potentially important connections. Full connectome analyses will allow a more comprehensive characterization of amygdala network alterations.

## 5 CONCLUSIONS

In summary, this study demonstrated distinct patterns of amygdala structural alterations in both major white matter fiber bundles and direct region‐to‐region connections across the canonical FTD syndromes. We also identified subtype‐specific associations between amygdala connectivity and social cognitive deficits, providing a plausible neural basis for the behavioral disturbances characteristic of these syndromes. Together, these findings not only expand our knowledge of amygdala integrity in FTD but may also, in time, assist in the development of interventions tailored to each FTD subtype.

## CONFLICT OF INTEREST STATEMENT

The authors declare no conflicts of interest. Author disclosures are available in the Supporting Information.

## CONSENT STATEMENT

All participants provided informed consent to take part in the study.

## Supporting information



Supporting Information

Supporting Information
