# Supplementary material for: Altered amygdala structural connectivity and relations to social cognition in frontotemporal dementia
Source: Alzheimers Dement. 2026 May 18;22(5):e71482. doi: 10.1002/alz.71482 (PMC13183591; doi:10.1002/alz.71482)
Supplement: Supplementary file 1 — Supporting Information [file ALZ-22-e71482-s002.pdf]

Cortical thickness analysis

Methods

Cortical reconstruction and volumetric segmentation of the T1s was performed with FreeSurfer "recon-all" pipeline [1]. Gyral and sulcal units were parcellated [2,3], to create maps of curve and sulcal depth. Cortical thickness was then calculated as the closest distance between the grey/CSF and grey/white boundaries at each vertex on the surface of the brain [1]. Cortical thickness was smoothed using a 15 mm full-width at half-height Gaussian kernel to minimize the effects of imperfect cortical alignment and enhance the signal-to-noise ratio [4].

For each patient group, a whole-cortex between-group comparison against healthy controls was performed separately for the left and right hemispheres using a vertex-wise GLM. To control family-wise error across the cortical surface, cluster-wise inference was performed using nonparametric permutation testing [5]. For each hemisphere and contrast, 1,000 permutations were run using a two-sided (absolute-value) cluster-forming threshold of $p < 0.001$, and results were considered significant at a cluster-wise corrected threshold of $p < 0.05$.

Results

The pattern of cortical thinning in each patient group is consistent with the canonical atrophy patterns of these syndromes (**Supplementary Fig. 1**).

BvFTD showed the most extensive cortical changes compared to controls, involving bilateral frontal and temporal regions, such as the orbitofrontal and inferior frontal cortices, anterior cingulate, insula, and temporal cortices.

SD showed bilateral, left-greater-than-right temporal cortical thinning, involving the superior, middle, and inferior temporal gyri and temporal pole, with extension into ventral temporal regions including the fusiform and parahippocampal cortex, as well as additional involvement of the insula and orbitofrontal cortex.

In PNFA, cortical thinning was also left-lateralised, particularly affecting the inferior frontal gyrus, including the pars opercularis and pars triangularis, with extension into the precentral gyrus and rostral middle frontal cortex, and additional involvement of the paracentral region and superior frontal gyrus.

Supplementary Figure 1. Cortical thickness reduction in FTD groups compared to controls

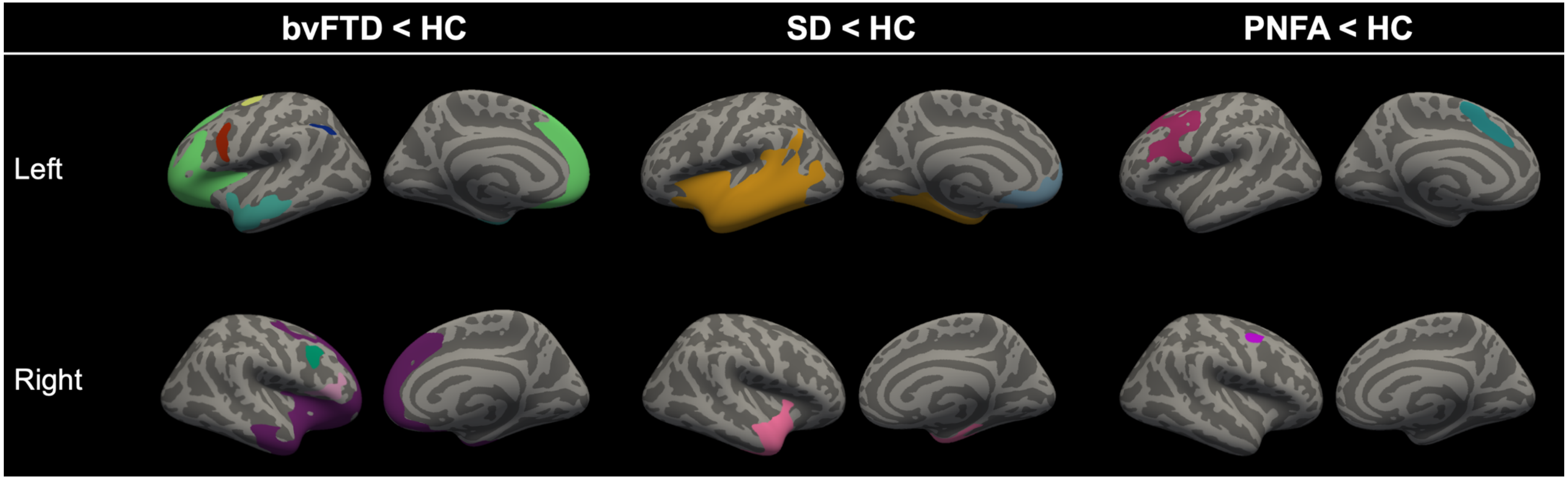

Coloured regions indicate regions of significantly reduced cortical thickness in each patient group compared to healthy controls, as denoted by "<" ($P < 0.001$).

bvFTD = behavioural-variant frontotemporal dementia; HC= healthy controls; PNFA= progressive nonfluent aphasia; SD= semantic dementia.

Supplementary Figure 2. Fibre density and cross-section (FDC) reductions in amygdala-associated tracts in FTD subtypes compared to controls

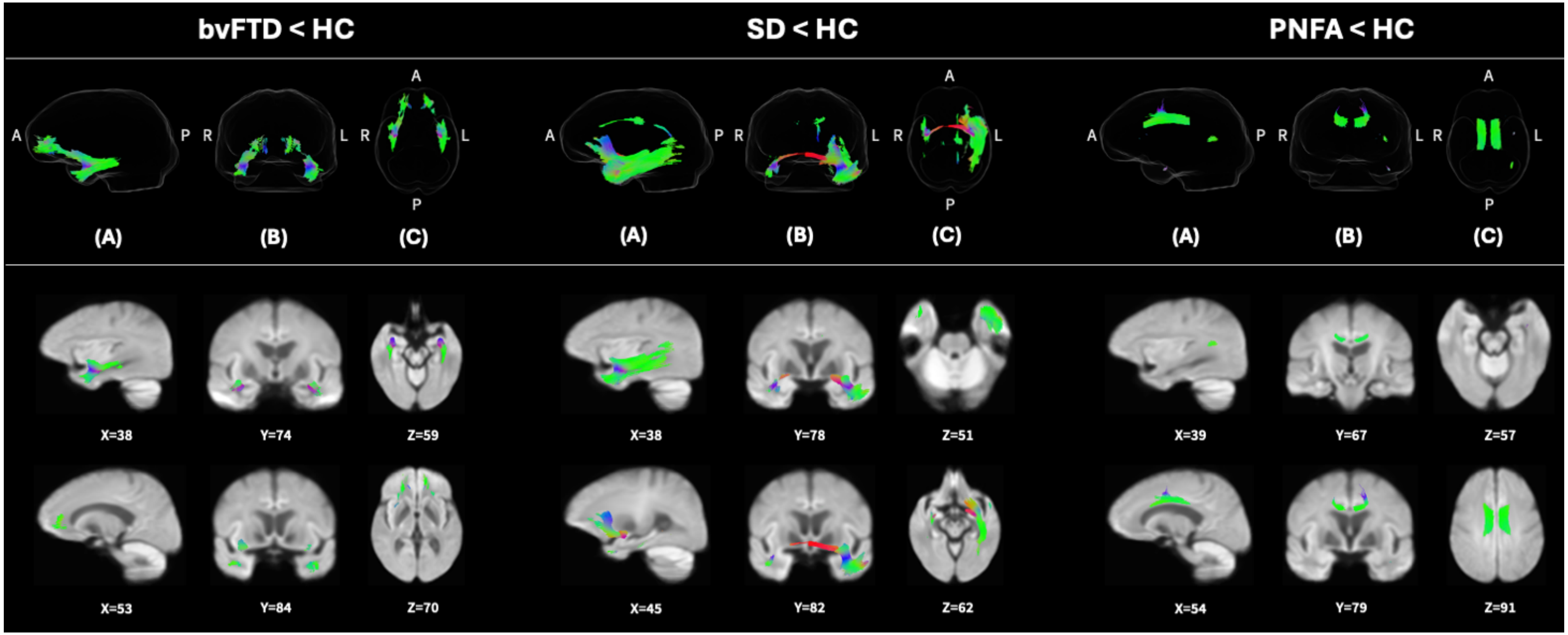

Coloured streamlines indicate regions of significant FDC reductions in each FTD subtype compared to controls (family-wise error corrected $P < 0.05$) across all amygdala-associated tracts (anterior commissure, cingulum, inferior fronto-occipital fasciculus, inferior longitudinal fasciculus, uncinate fasciculus). Streamlines are colour-coded by fibre tract direction: green=anterior-posterior; red=medial-lateral; blue=superior-inferior. Panels display (A) sagittal, (B) coronal, and (C) axial views, with coordinates (X, Y, Z) reported in MNI space.

A= anterior; bvFTD= behavioural variant frontotemporal dementia; HC= healthy controls; I= inferior; L= left; P= posterior; PNFA= progressive nonfluent aphasia; R= right; S= superior; SD= semantic dementia

Supplementary Table 1 Mean connectivity strengths (SIFT2 weights) between the amygdala and its top ten connected brain regions in FTD patients and controls.

	bvFTD n=21	SD n=19	PNFA n=18	HC n=28	Group Effect	*P*-value	*Post hoc* tests
**Temporal pole**							
Left	21446.21 ± 7555.62	15494.77 ± 8859.96	22724.02 ± 5248.29	22019.80 ± 3599.60	4.550	0.006	SD < bvFTD*, PNFA*, HC*
	(18006.93, 24885.49)	(10773.63, 20215.91)	(19817.61, 25630.42)	(20595.85, 23443.76)			
Right	25722.86 ± 6996.25	27763.18 ± 5339.37	28745.93 ± 4515.07	29262.23 ± 7820.29	1.225	-	-
	(22538.20, 28907.51)	(24806.33, 30720.02)	(26245.57, 31246.29)	(26229.84, 32294.62)			
**Entorhinal cortex**							
Left	13183.03 ± 6202.93	8467.46 ± 5659.45	19296.58 ± 5569.62	19219.41 ± 7016.15	12.223	< 0.001	bvFTD < PNFA*, HC*;
	(10279.97, 16086.09)	(5653.08, 11281.84)	(15930.89, 22662.27)	(15837.74, 22601.09)			SD < PNFA**, HC**
Right	13372.15 ± 5962.59	12630.97 ± 6492.27	18877.01 ± 6079.34	18570.91 ± 5829.79	4.996	0.004	bvFTD, SD < HC*
	(10581.57, 16162.73)	(9171.49, 16090.46)	(14792.85, 22961.17)	(15986.13, 21155.7)			
**Putamen**							
Left	12775.70 ± 6682.53	10673.19 ± 3422.94	13790.95 ± 4890.00	14464.17 ± 6579.45	3.928[a]	-	-
	(9733.85, 15817.55)	(8696.85, 12649.54)	(11276.74, 16305.15)	(11748.3, 17180.03)			
Right	9607.97 ± 4371.26	9298.45 ± 5406.84	8371.60 ± 3527.33	13383.59 ± 4301.33	5.618	0.002	bvFTD, SD, PNFA < HC*
	(7618.2, 11597.74)	(6609.69, 11987.21)	(6617.50, 10125.70)	(11608.10, 15159.08)			
**Thalamus**							
Left	10126.61 ± 3182.01	12680.31 ± 3565.51	12667.10 ± 3990.47	13475.37 ± 2688.84	4.221	0.008	bvFTD < HC*
	(8678.18, 11575.04)	(10847.10, 14513.53)	(10540.73, 14793.47)	(12365.47, 14585.27)			
Right	9641.43 ± 3580.40	10527.01 ± 3107.59	10727.75 ± 3374.37	12464.51 ± 3358.93	2.869	0.042	bvFTD < HC*
	(8011.65, 11271.21)	(9029.20, 12024.82)	(8859.09, 12596.42)	(11078.01, 13851.01)			
**Insula**							
Left	6181.26 ± 2541.01	6432.75 ± 4196.08	6066.30 ± 2712.98	7630.29 ± 3170.12	1.131	-	-
	(4956.53, 7405.98)	(4410.31, 8455.19)	(4717.17, 7415.43)	(6321.73, 8938.85)			
Right	7348.62 ± 3407.72	7994.70 ± 2915.14	6443.70 ± 2616.64	9494.07 ± 3684.09	3.554	0.018	PNFA < HC*
	(5797.44, 8899.79)	(6545.03, 9444.36)	(5142.48, 7744.93)	(8036.69, 10951.44)			

**Hippocampus**							
Left	6017.93 ± 3079.18	4892.33 ± 3934.13	7453.24 ± 3052.36	9855.52 ± 3111.04	24.463[a]	< 0.001	bvFTD, SD < HC**
	(4616.30, 7419.55)	(2996.14, 6788.52)	(5935.33, 8971.14)	(8649.19, 11061.86)			
Right	5235.67 ± 3102.88	4717.92 ± 1808.13	6126.74 ± 2823.05	7930.28 ± 2815.25	17.170[a]	< 0.001	bvFTD, SD < HC*
	(3823.25, 6648.08)	(3846.43, 5589.41)	(4722.87, 7530.61)	(6793.18, 9067.39)			
**Pallidum**							
Left	3715.22 ± 2490.00	5188.79 ± 3695.00	3923.74 ± 2225.79	6675.87 ± 3688.94	11.435[a]	0.010	bvFTD, PNFA < HC*
	(2581.79, 4848.66)	(3407.85, 6969.72)	(2816.88, 5030.60)	(5185.87, 8165.86)			
Right	7669.66 ± 4559.36	6205.76 ± 3752.81	6568.64 ± 2870.44	8059.75 ± 3879.04	3.421[a]	-	-
	(5594.27, 9745.06)	(4339.53, 8071.98)	(5141.21, 7996.08)	(6492.97, 9626.53)			
**Cerebellum cortex**							
Left	4721.31 ± 1909.34	5630.81 ± 2873.04	5127.17 ± 2898.60	7239.56 ± 3040.62	4.306	0.007	bvFTD < HC*
	(3852.19, 5590.44)	(4233.73, 7027.89)	(3948.36, 6305.99)	(6036.73, 8442.39)			
Right	5260.20 ± 2787.53	6031.70 ± 2804.52	5350.47 ± 2198.58	7485.63 ± 3354.59	3.077	0.032	-
	(3991.34, 6529.07)	(4679.97, 7383.44)	(4257.14, 6443.80)	(6158.60, 8812.66)			
**Fusiform gyrus**							
Left	4900.49 ± 2753.02	4067.67 ± 3328.34	5418.09 ± 2591.07	5997.47 ± 3498.25	5.111[a]	-	-
	(3647.33, 6153.65)	(2463.47, 5671.88)	(4085.88, 6750.29)	(4640.99, 7353.95)			
Right	5549.32 ± 2793.30	3990.22 ± 2863.90	5815.30 ± 3380.06	6002.15 ± 2712.70	2.003	-	-
	(4277.82, 6820.81)	(2609.86, 5370.57)	(4134.43, 7496.16)	(4929.04, 7075.26)			
**Inferior temporal gyrus**							
Left	3961.81 ± 2443.97	2256.98 ± 1364.90	5347.16 ± 2955.10	5938.12 ± 2783.63	8.854	< 0.001	SD < PNFA*, HC**
	(2818.00, 5105.63)	(1578.23, 2935.72)	(3877.62, 6816.69)	(4858.74, 7017.50)			
Right	5543.43 ± 3511.42	2857.48 ± 2202.20	4842.47 ± 2441.05	5738.23 ± 2440.03	4.956	0.003	SD < bvFTD*, HC*
	(3900.03, 7186.82)	(1796.05, 3918.91)	(3628.56, 6056.37)	(4792.09, 6684.38)			

Values are mean ± standard deviation (95% confidence intervals).

[a] Kruskal-Wallis H test.

*P < 0.05; **P < 0.001.

bvFTD= behavioural variant frontotemporal dementia; HC= healthy controls; PNFA= progressive nonfluent aphasia; SD= semantic dementia.

References

1. Fischl B, Dale AM. Measuring the thickness of the human cerebral cortex from magnetic resonance images. *Proc National Acad Sci*. 2000;97(20):11050-11055. doi:10.1073/pnas.200033797

2. Desikan RS, Ségonne F, Fischl B, et al. An automated labeling system for subdividing the human cerebral cortex on MRI scans into gyral based regions of interest. *NeuroImage*. 2006;31(3):968-980. doi:10.1016/j.neuroimage.2006.01.021

3. Fischl B, Kouwe A van der, Destrieux C, et al. Automatically Parcellating the Human Cerebral Cortex. *Cereb Cortex*. 2004;14(1):11-22. doi:10.1093/cercor/bhg087

4. Lerch JP, Evans AC. Cortical thickness analysis examined through power analysis and a population simulation. *NeuroImage*. 2005;24(1):163-173. doi:10.1016/j.neuroimage.2004.07.045

5. Greve DN, Fischl B. False positive rates in surface-based anatomical analysis. *NeuroImage*. 2018;171:6-14. doi:10.1016/j.neuroimage.2017.12.072
